# Retrocrural Space Involvement on Computed Tomography as a Predictor of Mortality and Disease Severity in Acute Pancreatitis

**DOI:** 10.1371/journal.pone.0107378

**Published:** 2014-09-15

**Authors:** Haotong Xu, Lukas Ebner, Shiming Jiang, Yi Wu, Andreas Christe, Shaoxiang Zhang, Xiaoming Zhang, Zhulin Luo, Fuzhou Tian

**Affiliations:** 1 Postdoctoral Workstation, the General Surgery Center of the Peoples’ Liberation Army, Chengdu Army General Hospital, Chengdu, Sichuan, P. R. China; 2 Department of Radiology, Sichuan Provincial People’s Hospital Supo, Chengdu, Sichuan, P. R. China; 3 Department of Radiology, Inselspital, University of Bern, Freiburgstrasse, Bern, Switzerland; 4 Department of Radiology, Nanchong Central Hospital of North Sichuan Medical College, Nanchong, Sichuan, P. R. China; 5 Institute of Computing Medicine, Third Military Medical University, Chongqing, P. R. China; 6 Sichuan Key Laboratory of Medical Imaging, Department of Radiology, Affiliated Hospital of North Sichuan Medical College, Nanchong, Sichuan, P. R. China; Klinikum rechts der Isar der TU München, Germany

## Abstract

**Background:**

Because computed tomography (CT) has advantages for visualizing the manifestation of necrosis and local complications, a series of scoring systems based on CT manifestations have been developed for assessing the clinical outcomes of acute pancreatitis (AP), including the CT severity index (CTSI), modified CTSI, etc. Despite the internationally accepted CTSI having been successfully used to predict the overall mortality and disease severity of AP, recent literature has revealed the limitations of the CTSI. Using the Delphi method, we establish a new scoring system based on retrocrural space involvement (RCSI), and compared its effectiveness at evaluating the mortality and severity of AP with that of the CTSI.

**Methods:**

We reviewed CT images of 257 patients with AP taken within 3–5 days of admission in 2012. The RCSI scoring system, which includes assessment of infectious conditions involving the retrocrural space and the adjacent pleural cavity, was established using the Delphi method. Two radiologists independently assessed the RCSI and CTSI scores. The predictive points of the RCSI and CTSI scoring systems in evaluating the mortality and severity of AP were estimated using receiver operating characteristic (ROC) curves.

**Principal Findings:**

The RCSI score can accurately predict the mortality and disease severity. The area under the ROC curve for the RCSI versus CTSI score was 0.962±0.011 versus 0.900±0.021 for predicting the mortality, and 0.888±0.025 versus 0.904±0.020 for predicting the severity of AP. Applying ROC analysis to our data showed that a RCSI score of 4 was the best cutoff value, above which mortality could be identified.

**Conclusion:**

The Delphi method was innovatively adopted to establish a scoring system to predict the clinical outcome of AP. The RCSI scoring system can predict the mortality of AP better than the CTSI system, and the severity of AP equally as well.

## Introduction

In addition to the pathological changes of the pancreas itself from acute pancreatitis (AP), various local complications of AP may appear in the retroperitoneal space or the structures around the pancreas. These complications involve fluid collections, pseudocyst, peripancreatic abscess and vascular disorders, etc. The complications around the pancreas, especially those in the retroperitoneal space, will progress to severe AP, and can lead to a poor prognosis [1].

The diagnostic imaging modalities for AP have a significant role in confirming the diagnosis of the disease, helping detect the extent of pancreatic necrosis, and diagnosing local complications [2]. Because of their advantages for visualizing the manifestation of necrosis and local complications, CT and magnetic resonance imaging (MRI) have been used in assessing the clinical outcomes of patients with AP. A series of scoring systems and prediction methods based on imaging manifestations have been developed for this purpose, including the CT severity index (CTSI), modified CTSI, extrapancreatic inflammation on CT (EPIC), and MR severity index (MRSI) [3], [4], [5], [6].

The CTSI, developed by Balthazar and colleagues in 1994 [3], was a significant advance in assessing the clinical outcomes of patients because it helped clinicians to discriminate among mild, moderate, and severe forms of pancreatitis. The CTSI focuses on the presence and degree of pancreatic inflammation and necrosis. On a 10-point severity scale, points are awarded for the presence or absence of fluid collections, in combination with an assessment of the presence and degree of pancreatic necrosis [3]. Although this system has been successfully used to predict overall morbidity and mortality in patients with AP, we found limitations in the application of the CTSI scoring system. Observers often diverged from each other in counting the locations for fluid collections due to the development of the radiological anatomy of the retroperitoneal space. Subsequently, the inter-rater agreement for scoring CT scans using the CTSI may be poor.

Mortelé et al. have indicated other shortcomings of the CTSI, such as no correlation with extrapancreatic parenchymal complications, and no significant difference in mortality between 30 and 50% and more than 50% necrosis [7], [8]. Thus, researchers have attempted to establish a new scoring systems - the modified CTSI, which incorporates extrapancreatic complications into the CTSI, and the EPIC, which is based exclusively on the presence of systemic inflammation (including pleural effusion, ascites, and retroperitoneal inflammation) [4], [5]. Although some comparative studies have confirmed that the predictive value of these new scoring systems was better than that of the CTSI, the effective forecasting method has not been applied in constructing these scoring systems.

Originally developed as a systematic, interactive forecasting method that relies on a panel of experts [9], the Delphi method is a structured communication technique based on the principle that forecasts from a structured group of individuals are more accurate than those from unstructured groups [10]. Experts answer questionnaires in two or more rounds. After each round, a facilitator provides an anonymous summary of the experts’ forecasts from the previous round as well as the reasons they provided for their judgments. Thus, the experts are encouraged to revise their earlier answers in light of the replies from other members of their panel. It is believed that during this process, the range of the discrimination will decrease and the group will converge towards a consensus. The process is stopped after a pre-defined stop criterion (e.g. number of rounds, achievement of consensus); namely, the experts will agree with all items of the scoring system in the final round. In 1999, Ricke et al. used a Delphi consensus procedure to establish guidelines for standardized diagnostic imaging of neuroendocrine tumors [11].

Calculating the accuracy of a diagnostic test using standard definitions (such as CTSI) unavoidably includes the risk of bias in some circumstances. Receiver operating characteristic (ROC) analysis offers the possibility of bias-correction by which the most appropriate cutoff value can be determined [12]. Moreover, the area under the ROC curve is a reliable measure of overall predictive discrimination [13]. Thus, ROC analysis should be the preferred method for assessing the predictive value of imaging techniques [5].

In our previous research employing the Visible Human Project and CT images to study the draining pathways of peripancreatic fluid to the mediastinum in recurrent AP, we discovered that most of the pancreatic fluid might first enter the retrocrural space on its transdiaphragmatic passage into the mediastinum, except for the fluid in the right posterior mediastinum [14]. The retrocrural space is the sentinel mediastinal space involved in AP. Therefore, our purpose was the following: (1) to observe the CT manifestations of RCSI in AP, RCSI is the infectious conditions involving the bilateral crus of diaphragm, the mediastinal pleura, the retrocrural space itself and the adjacent pleural cavity; (2) to establish the RCSI scoring system based on the Delphi method; and (3) to use ROC analysis of the RCSI score in predicting the mortality and disease severity in patients with AP, and compare the RCSI score with the CTSI score.

## Materials and Methods

### 1 Ethics statement

This study was approved by the Ethics Review Board of the Chengdu Army General Hospital. The second Chinese Visible Human (CVH2) dataset was from a voluntary donation and was approved for medical research [15]. Obtaining CT scans of patients with AP was approved by the Institutional Review Board of our Hospital. Written informed consent was obtained from the patients before CT scans, and no identifiable information (i.e., age and gender) was reported in this study.

### 2 Patients

Medical records and CT images were reviewed retrospectively for consecutive patients with AP admitted to our hospital between January and December 2012. Patients were diagnosed with AP using the International Classification of Diseases, Ninth Revision, Clinical Modification code for AP (577.0) [16]. Eligibility criteria for patients in this study were: (1) in-patient, (2) acute onset of symptoms, (3) pancreatitis at first onset, (4) abdominal CT scans, with scanned coverage from the diaphragmatic dome to the iliac crest, and (5) CT examinations obtained 3–5 days after admission. Exclusion criteria in this study were: (1) history of traumatic pancreatitis or postoperative pancreatitis, (2) history of laparotomy or a previous hospitalization for AP that might hinder the interpretation of the severity of AP, and (3) without contrast-enhanced CT scans because of contraindication to iodinated contrast medium and the potential risk of nephrotoxicity.

A total of 257 patients, including 196 men and 61 women, with a mean age of 51.2±12.3 years (range 23–89 years), was recruited for this study. The etiologies of AP were gallstones in 92 patients, alcohol abuse in 71, gallstones and chronic alcohol abuse in 55, post-endoscopic retrograde cholangiopancreatography inflammation in 17, and miscellaneous or uncertain origin in 22.

### 3 Clinical observation

Clinical outcome was recorded as: (1) severe AP, diagnosed according to the Atlanta classification system [17]; and (2) systemic disease (intensive care unit admission [ICU] or mortality). Percutaneous CT-guided catheter drainage and/or surgical procedures were performed in patients who had pseudocysts at follow-up examinations, infected pancreatic necrosis and in those with abdominal compartment syndrome.

### 4 Abdominal CT scans

All patients underwent CT scans on a 64-slice scanner (GE Healthcare, Milwaukee, WI, USA) in our hospital. The CT parameters were 120 kVp, 260 mAs, 8×1.25-mm detector configuration, 10.0-mm beam collimation, 18.75 mm/rotation table speed, 5.0-mm section thickness, 0.5-s gantry rotation time, and standard reconstruction algorithm. Unenhanced CT scans were done first, then contrast material (Iopamiron 300, Schering, Berlin, Germany) was administered intravenously to all patients at a flow rate of 3–5 ml/s. The scans extended from the diaphragmatic dome to the iliac crest. If an abnormality was observed on the highest or lowest section, additional images were obtained after the enhanced scan; especially when the RCSI or the combined interfascial plane involved was shown on CT images.

### 5 Anatomic observation of the retrocrural space

**Figure 1:**
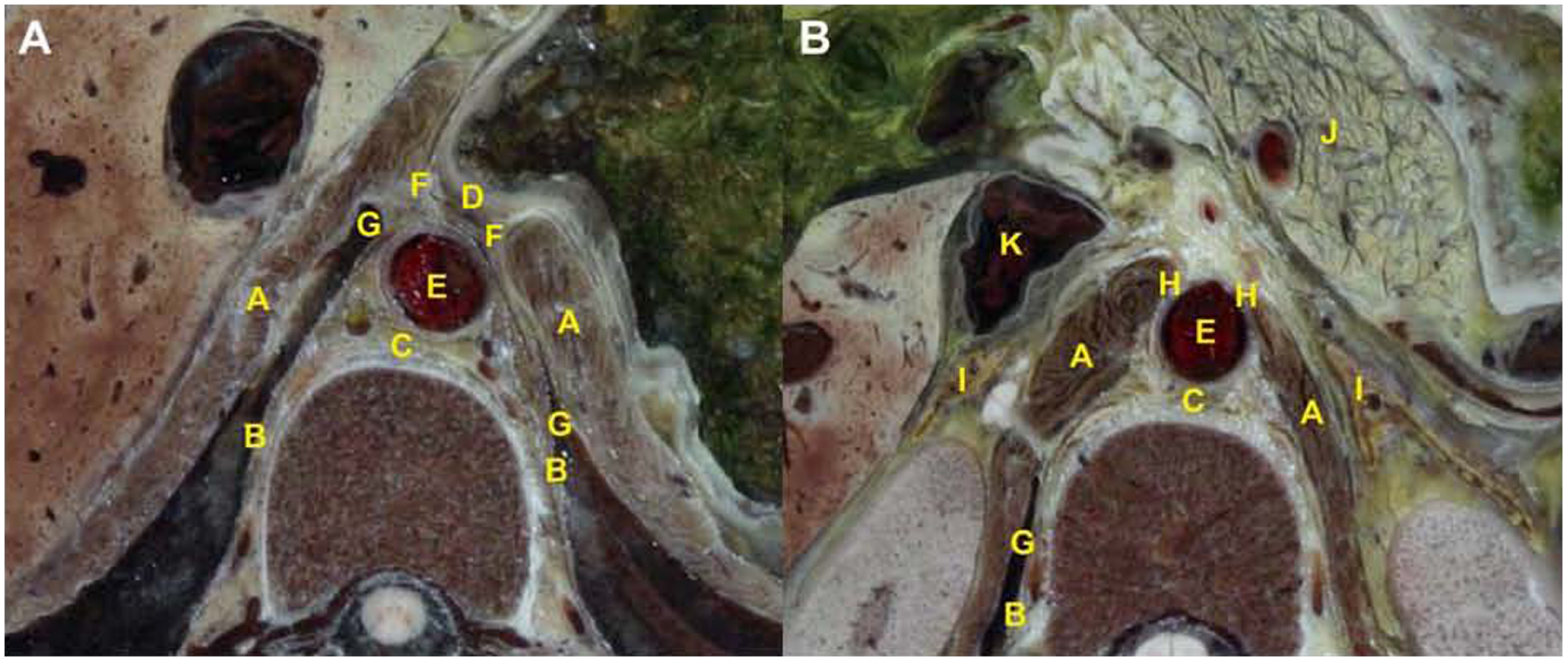
Visualization of the anatomic location of retrocrural space across diaphragmatic hiatuses section on CVH2. (**A**) On the upper section, the anterior margin of the retrocrural space (C) consists of the distal esophagus (D), the posterior border is thoracolumbar vertebra, both anterolateral borders are composed of the diaphragmatic crus (A), both posterolateral borders are made up of the mediastinal pleura (B). The aorta (E) is situated in the retrocrural space. The potential recess located between the diaphragmatic crura and the mediastinal pleura is the interior costophrenic sulcus (G). The peripancreatic fluid may drain into the retrocrural space via the esophageal hiatus (F). (**B**) On the lower section, the anterior border of the retrocrural space is open to the retroperotoneum, the posterior border is the lumbar vertebra, both lateral borders are made up of the diaphragmatic crus (A), the mediastinal pleura (B) constitutes the right posterolateral border. The inferior vena cava (K) and the both sides of adrenal glands (I) are distributed at the anterolateral direction of the retrocrural space. Furthermore, the peripancreatic fluid that originates from the pancreas (J) may drain into the retrocrural space across the aortic hiatus (H).

The anatomic features of the retrocrural space were observed on thin-slice cross-sectional images derived from the CVH2 dataset [15]. In consecutive cross-sectional images, the retrocrural space can be defined as a triangular region that represents the most inferior portion of the posterior mediastinum, with no distinct border with the retrocardiac space of the posterior mediastinum in the cranial direction. We defined the appearance of the esophageal hiatus as the first section of the retrocrural space on consecutive cross sections. The point at which the right crura adheres to the lateral surface of the third lumber vertebral body is the inferior border of the retrocrural space. Except for the diaphragmatic hiatuses, the space is delineated anteriorly and laterally by the diaphragmatic crura in the anteroinferior direction, and the mediastinal pleura in the posterosuperior direction. Posteriorly, boundaries include the ventral aspect of the thoracolumbar vertebra (Fig. 1).

### 6 Establishment of RCSI scoring system based on the Delphi process

To begin, we clarify definitions in the RCSI scoring system. First, normal pleura cannot be imaged on CT scans. Mediastinal pleura that can be visualized indicates thickening of the pleura [18], [19]. Second, hydropsia of the diaphragm indicates fluid distribution. Partial swelling in these areas attenuates the CT values [18], [20]. Third, haziness and a streaky density in the retrocrural space indicate infectious conditions involving this space similar to the gastric bare area involved in AP [21]. Fourth, we have confirmed that the peripancreatic fluid might extend into the retrocrural space only via esophageal or aortic hiatus in AP [14]. Fifth, pleural effusions, which could be symptomatic in mediastinal fluid collections or result from the development of a fistulous tract into the pleural cavity, might be present during the second stage of the pulmonary complications of AP [22]. As an important item, the “pleural effusion” was adopted in the RCSI scoring system. Sixth, if the single crura and/or the partial retrocrural space at the posterointernal direction of the homolateral crura are involved in AP, it means unilateral involvement. When both sides of the crura and/or the bilateral sides of the retrocrural space are involved simultaneously, it means bilateral involvement.

To date, the Delphi method has been used widely to establish index systems and to identify a specific index. A Delphi seminar aimed at establishing an RCSI scoring system in evaluating the mortality and disease severity in AP was held on November 5, 2011. A multidisciplinary panel of 12 medical experts was selected to take part in the Delphi process. The criteria for inclusion on the panel were radiological and clinical experts with at least 20 years of experience in AP, and anatomical experts with at least 15 years of experience in sectional anatomy. Prior to the conference, all the experts were provided with recent radiological research reports of pathologic conditions that involved or invaded the retrocrural space, and a radiological scoring system for evaluating the severity of AP, besides of the CTSI, the modified CTSI, the MRSI [3], [4], [6], [23], [24], [25]. The updated results of the discussion were given to all experts during the initial two rounds of the Delphi procedure. The feedback served as a basis for discussion in the subsequent round. Finally, in the third round, all experts in the panel reached a consensus on a scoring system for RCSI.

### 7 Quantification of RCSI and CTSI scores on CT

All CT examinations were reviewed by two raters with 10 and 16 years of experience in interpreting abdominal CT images, who were blinded to the laboratory data and clinical outcome. CTSI scores were calculated for every patient (Tab. 1). RCSI scores were quantified for every patient, based on the presence of CT manifestations of RCSI (Tab. 2).

**Table 1:** CTSI scoring system on CT scans in AP.

Prognostic Indicator	Score
Pancreatic inflammation	
Normal pancreas	0
Focal or diffuse enlargement of the pancreas	1
Intrinsic pancreatic abnormalities withinflammatory changes in peripancreatic fat	2
Single, ill defined fluid colletion or phlegmon	3
Two or more poorly defined collectionsor presence of gas in or adjacent to pancreas	4
Pancreatic Necrosis	
None	0
≤30%	2
30–50%	4
>50%	6

**Table 2:** RCSI scoring system on CT scans in AP.

Prognostic indicator	Score^b^
Hydropsia and rough edge of thediaphragm outside the retrocrural space	1
Thickening of the mediastinalpleura outside the retrocrural space	1
Haziness and streaky densityin the retrocrural space	1
Fluid extends into the retrocruralspace across esophageal or aortic hiatus^a^	1
Pleural effusion	2

a If fluid extends into the retrocrural space across both hiatuses, the score should be 2. ^b^If CT manifestations shows bilateral involvement, the score should be doubled.

### 8 Statistical analysis

Results of the RCSI and CTSI scores were given as the mean of the two raters. Inter-rater agreement for the RCSI and CTSI scores was tested using the kappa statistic, which was used to estimate the proportion of inter-rater agreement above that expected by chance. A weighted kappa statistic of 0.41–0.60 was considered moderate agreement, 0.61–0.80 good agreement, and 0.81–1.00 excellent agreement.

ROC analysis was also performed to examine the predictive effect of RCSI and CTSI scoring on the mortality and severity of AP, using mortality and severe AP as the dependent variables. The discriminative powers of the RCSI and CTSI scoring systems were visualized using ROC curves, including the area under the curve (AUC), with 95% confidence interval (CI). Additionally, the AUC values of the two scoring systems were compared using the z-test. The best cutoff values on the ROC curves of the RCSI and CTSI scores were calculated based on the maximum Youden index.

Statistical analysis was performed using commercially available software (SPSS 13.0 version, Chicago, IL, USA), except for the comparison of the AUC of the two scoring systems, which was done with MedCalc 11.6 (MedCalc Software, Mariakerke, Belgium).

## Results

### 1 Patient Outcome

Of the 257 patients surveyed, 60 (23.3%) were admitted to the ICU. A total of 166 patients (64.6%) were diagnosed as having severe AP; 134 patients (52.1%) recovered with medical treatment alone and were discharged, while 123 patients (47.9%) underwent surgical and/or percutaneous interventions. Percutaneous CT-guided catheter drainage of peripancreatic fluid collections was performed in 68 patients (26.5%), local irrigation to the lesser sac in 52 patients and the drainage for a pseudocyst in 16 patients (6.2%). Twelve patients (4.7%) underwent surgical decompression for abdominal compartment syndrome. Moreover, 43 patients (16.7%) underwent a combination of procedures (necrosectomy and drainage) for the infected pancreatic necrosis. Fourteen patients (5.4%) with sepsis died. Eleven patients (4.3%) with alimentary tract hemorrhages died, and 48 patients (18.7%) died of multiple organ failure.

### 2 CTSI score

Pancreatic enlargement was seen in 12 patients (4.7%), pancreatic and/or peripancreatic fat inflammation was detected in 103 patients (40.1%), a single peripancreatic fluid collection was shown in 59 patients (23.0%), and two or more fluid collections and/or retroperitoneal air was imaged in 83 patients (32.3%); 73 patients (28.4%) did not have any necrosis; 87 patients (33.9%) exhibited imaging signs of necrosis in less than one-third of the pancreas; 58 patients (22.6%) demonstrated necrosis in one-third to one-half of the pancreas. Thirty-nine patients (15.2%) were diagnosed with necrosis in more than one-half of the pancreas.

### 3 RCSI score

**Figure 2:**
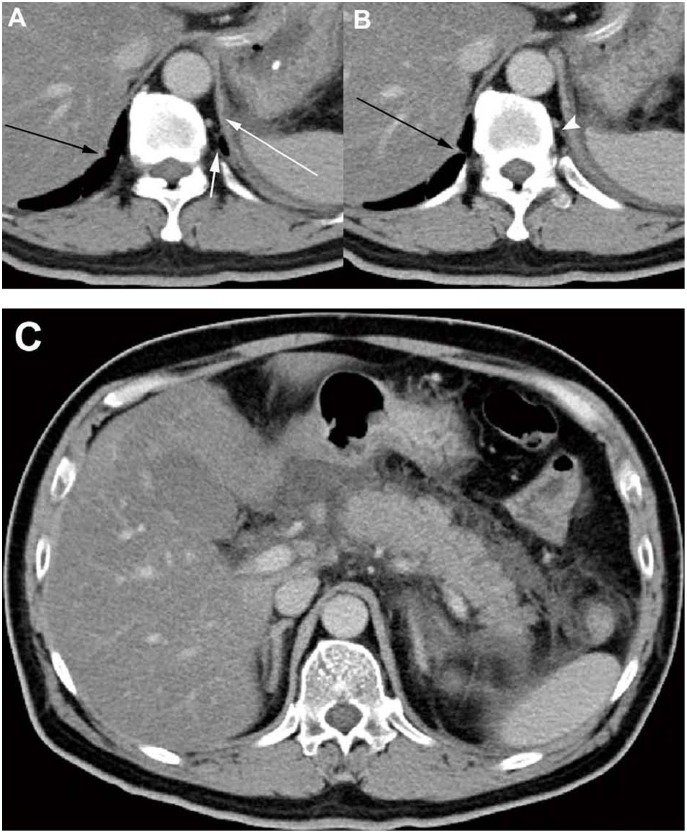
CT manifestations of the retrocrural space involvement in a 48-year-old man with AP. (**A**, **B**) CT scans obtained 3 days after admission showed acute fluid collections at the left subphrenic spaces. They resulted in the hydropsia of the left diaphragm (*long white arrow*), and the thickening of the left mediastinal pleura (*short white arrow*). In addition, a streaky density occurred at the left retrocrural space (*arrowhead*). On the opposite side, the right crus has a rough edge (*black arrow*). (**C**) CT scans displayed the peripancreatic fluid collections had extended into the left retroperitoneal space.

**Figure 3:**
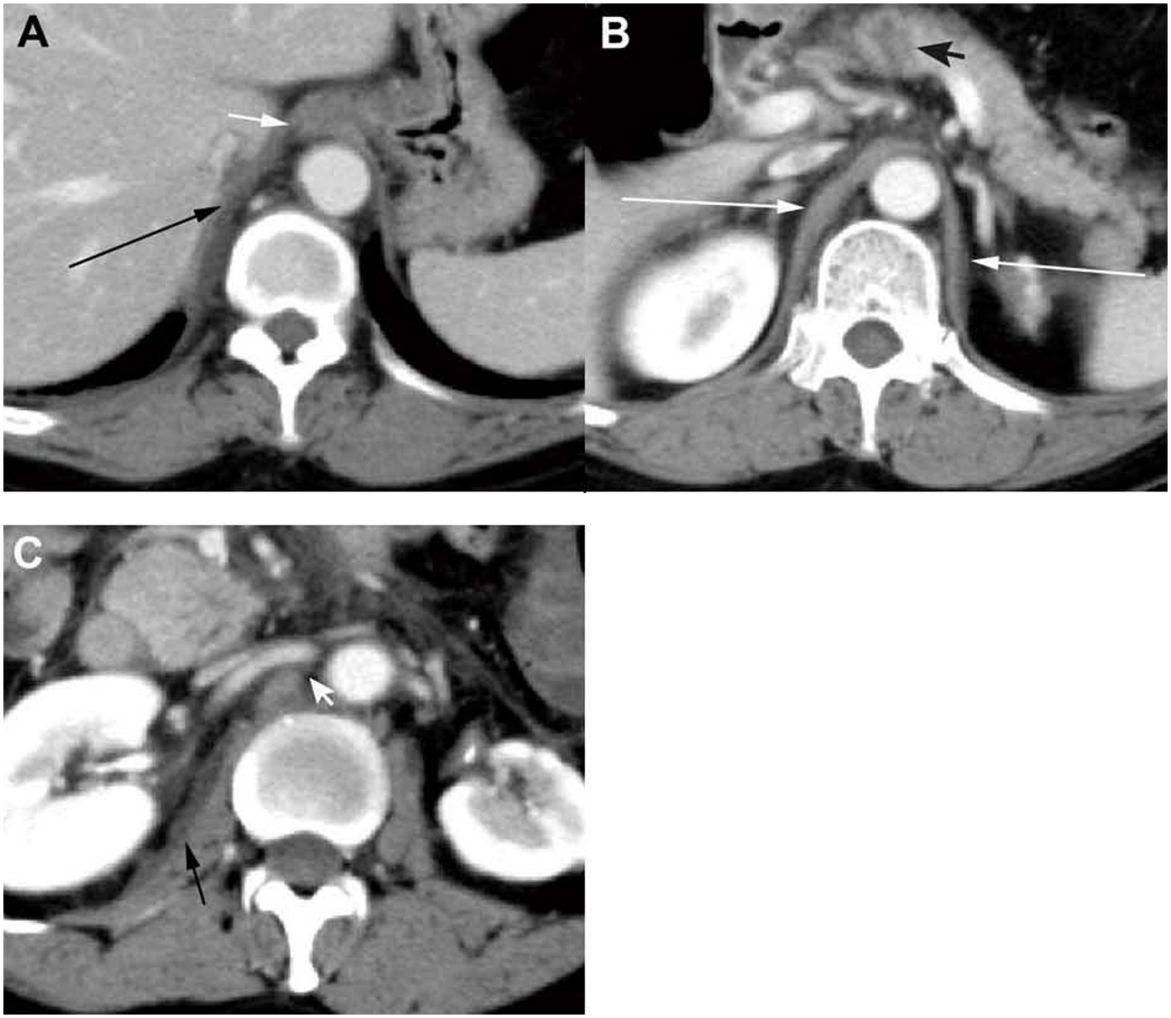
CT scans showing peripancreatic fluid draining into retrocrural space in a 46-year-old woman with AP. (**A**) On the upper section, CT scans obtained 4 days after admission showed the peripancreatic fluid had drained into the retrocrural space across the esophageal hiatus (*short white arrow*); then formed a fistulous tract into pleural cavity and developed into the pleural effusion (*long black arrow*). (**B**) On the middle section, pancreatic head necrosis is shown as a non-enhanced area that was less than 30% of total pancreatic area (*black arrowhead*). The bilateral diaphragmatic crus were shown hydropsia (*long white arrow*). (**C**) On the lower section, CT scans show the fluid had extended into the right retroperitoneal space (*short black arrow*) and further drained into the retrocrural space across the aortic hiatus (*white arrowhead*).

On the CT scans, one side or both sides of the diaphragmatic crura had been involved in 194 patients (75.5%) (Fig. 2A, B and 3B). Hydropsia and the rough edge of the mediastinal pleura were present in 123 patients (47.9%) (Fig. 2A). Haziness and streaky density in the retrocrural space were manifested in 105 patients (40.9%) (Fig. 2B). Pancreatic fluid had drained into the retrocrural space via the esophageal hiatus in 127 patients (49.4%), and via the aortic hiatus in 71 patients (27.6%) (Fig. 3A, C). One hundred fifty-four patients (59.9%) developed pleural effusion (Fig. 3A).

### 4 Comparison between RCSI and CTSI scores

The kappa statistics for the RCSI and CTSI scores between the two raters were 0.735±0.032 and 0.605±0.035, respectively. These values indicate good agreement for RCSI scores, but moderate agreement for CTSI scores; indicating RCSI scores are superior to CTSI scores. The mean RCSI score was 4.22±2.04 points (range 1–10) for all 257 patients, and the mean CTSI score was 5.26±2.58 points (range 1–10).

### 5 Predictive value of RCSI and CTSI scores for mortality

**Figure 4:**
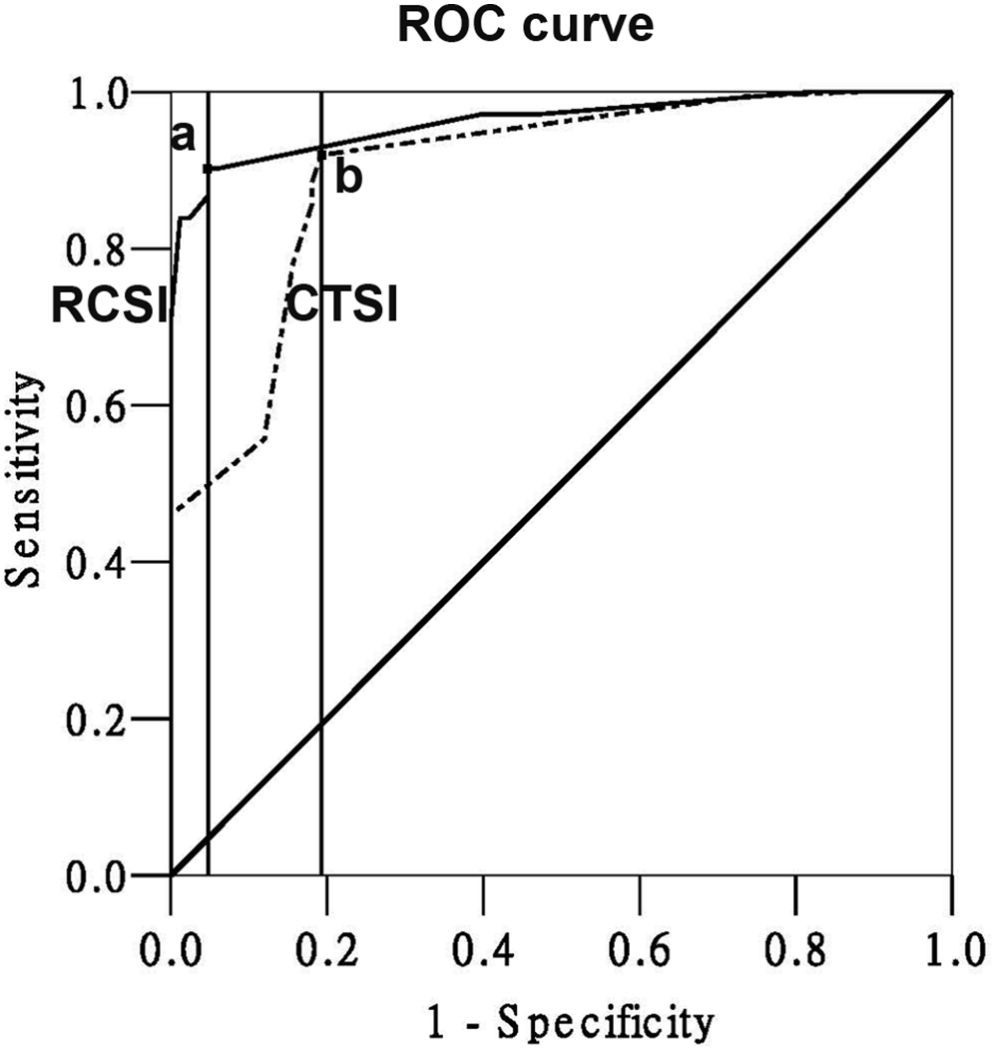
ROC curves of the RCSI score and CTSI score in predicting the mortality. (a: cutoff value = 4.0, sensitivity = 82.11%, specificity = 96.91%; b: cutoff value = 2.0, sensitivity = 77.27%, specificity = 91.12%).

The AUC for the RCSI score was 0.962 (95% CI 0.942–0.983), which shows the high accuracy of this index in predicting mortality (Fig. 4). An RCSI score of 4.0 was identified as the best cutoff value (maximum Youden index, YI = 0.854). The AUC for the CTSI score was 0.900 (0.859–0.940), with a best cutoff value of 2.0 (maximum YI = 0.727). The AUC of the RCSI score for the prediction of mortality was significantly higher than the AUC of the CTSI score (*P<*0.05, z-test). The sensitivity and specificity also showed that the RCSI score was superior to the CTSI score for predicting mortality (Tab. 3).

**Table 3:** Value of RCSI and CTSI scoring systems for predicting the mortality and severity of AP.

Scoring system	Cutoff value	Sensitivity	Specificity	PPV	NPV
RCSI (for MOR prediction)	4.0	82.11%	96.91%	93.98%	90.23%
CTSI (for MOR prediction)	2.0	77.27%	91.12%	81.93%	88.51%
RCSI (for SAP prediction)	3.0	79.38%	87.50%	84.62%	87.95%
CTSI (for SAP prediction)	4.0	81.91%	91.41%	84.62%	89.76%

MOR: mortality, SAP: severe acute pancreatitis, PPV: positive predictive value, NPV: negative predictive value.

### 6 Predictive value of RCSI and CTSI scores for severity of AP

**Figure 5:**
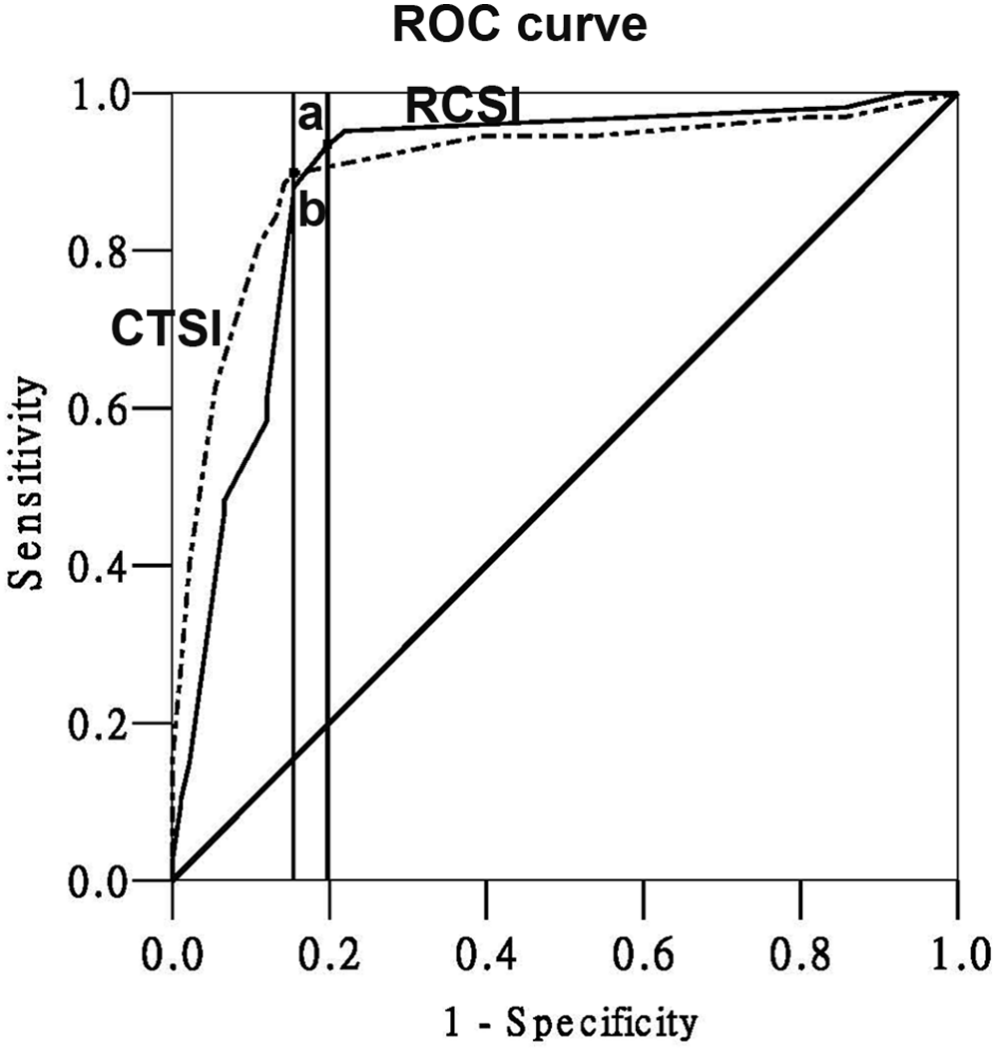
ROC curves of the RCSI and CTSI score in distinguishing between mild and severe AP. (a: cutoff value = 3.0, sensitivity = 79.38%, specificity = 87.50%; b: cutoff value = 4.0, sensitivity = 81.91%, specificity = 91.41%).

The AUC value for the CTSI score was 0.904 (0.864–0.944; Fig. 5). A CTSI score of 4.0 was identified as the best cutoff value (maximum YI = 0.744). The AUC value for the RCSI score was 0.888 (0.840–0.936) and the best cutoff value for the RCSI score was 3.0 (maximum YI = 0.736). There was no significant difference between the AUC of the CTSI score for predicting the severity of pancreatitis and the AUC of the RCSI score (*P>*0.001, z-test). The sensitivity and specificity, which were calculated to evaluate the diagnostic capacity of the two scoring systems for identifying the severity of pancreatitis, also indicated that there was no significant difference between the two scoring systems.

## Discussion

The present study is a follow-up to our previous research about draining pathways from the peripancreatic space to mediastinum in patients with AP [14]. These two studies show that transformation of the radiological anatomy to consequent CT manifestations can be used to predict prognosis. We investigated the RCSI scoring system to predict the patients’ prognosis and disease severity, and found that the RCSI score can accurately predict the mortality and disease severity in patients with AP. The RCSI score can substitute for the CTSI score to predict clinical outcomes of AP in some conditions; especially when the pelvis has not been included in the scanned coverage, but we can deduce that the fluid collections may occur in the pelvic extraperitoneal space due to the combined interfascial plane involved on CT images. Simultaneously, we should pay attention to the rarely anatomic variants and anomalies of the retrocrural space, because these variants may be mistaken for the CT manifestations of the RCSI [24]. For instance, the duplicated accessory diaphragm will probably be regarded as the hydropsia of the diaphragm.

The introduction of the CTSI in 1994 was a significant advance in the assessment of patients with AP [3]. Despite the CTSI having been successfully used to predict overall mortality in patients with AP, Balthazar and others have confirmed that when using the CTSI there was no significant difference in mortality between patients who have 30–50% necrosis and patients who have more than 50% necrosis [7], [26]. Recent publications have confirmed that the mediastinal acute fluid collection in patients with AP can result in increased morbidity and mortality [27]. Furthermore, as a risk factor for severe AP, pleural effusion was included in the RCSI scoring system [28]. We speculate that the presence of fluid drainage into the retrocrural space and pleural fluid may be responsible for the improved efficiency of prediction (Fig. 4), because they may be indicators of organ dysfunction. Our study also showed that an RCSI cutoff value of 4 was best for identifying the mortality rate. For an RCSI score higher than 4, clinicians should be alert to the possibility of further development of RCSI to dysfunction of one or more organs, which may lead to a fatal course of the disease.

An ideal prognostic method should have low inter-rater variability. The inter-rater variability of the RCSI score was lower than that of the CTSI score in our study (0.735±0.032 vs. 0.605±0.035). On the one hand, two observers often diverged from each other in counting the locations for fluid collections. Especially when the peripancreatic fluid involved both the left and right anterior pararenal space, or both the superior and inferior recess of the lesser sac in necrotizing pancreatitis, the two observers usually could not agree on whether to count it as one location or as two locations. It will influence the CTSI score. On the other hand, they are more familiar with the imaging of pathologic conditions that involve the retrocrural space than other radiologists because they have focused on the study of the radiological anatomy of the retroperitoneal space; they usually can reach an agreement on RCSI scores in a double-blinded condition.

Accompanied by the further study of the radiological anatomy of the retroperitoneal space [29], the concept of “interfascial planes” of the retroperitoneal space was suggested by Molmenti et al. in 1996 [30]. The study by Gore et al. confirmed the ability of interfascial planes to serve as spaces that can decompress retroperitoneal fluid collections and infiltrating diseases, especially when large volumes of fluid develop rapidly in severe AP [31]. Based on this concept, we suggest that these fascial planes be regarded as the locations for fluid collections when calculating the score for the CTSI.

Our study had some limitations. The RCSI scoring system was established based on the experience of experts, two radiologists came from our institute that focused on the study to the radiological anatomy of the retroperitoneal space, so some confounders were unavoidable. To control the confounders as much as possible, a multidisciplinary and anonymous panel of 12 experts was recruited, with one panelist being a radiological expert in image processing. However, the selected panelists were all local experts from southwest China; no international experts were included. Thus, whether this scoring system can be implemented in areas outside China requires further study. In order to build a rational consensus, we took some measures to assure that the process of collecting expert assessments is subject to the following principles: accountability, empirical control, neutrality, and fairness [32]. Although our study suggests that the predictive value of the RCSI scoring system for the mortality (AUC = 0.962±0.011) and severity of AP (AUC = 0.888±0.025) is valid, with low inter-rater variability (0.735±0.032), further study on the validity and reliability of this scoring system is necessary.

In summary, the main contribution of the present study is the establishment of a new scoring system, the RCSI, using the Delphi method. The RCSI can predict the mortality and severity of AP from initial CT scans. In addition, the RCSI is recommended for use as a supplement to the CTSI when we encounter the difficulty in calculating the CTSI score, especially when the pelvis has not been included in the scanned coverage.
